# Heat waves reveal additive genetic effects leading to sunburn resilience of grapevine berries

**DOI:** 10.3389/fpls.2025.1533345

**Published:** 2025-06-30

**Authors:** Tom Heinekamp, Franco Röckel, Katja Herzog, Oliver Trapp, Reinhard Töpfer, Florian Schwander

**Affiliations:** Julius Kuehn-Institut, Federal Research Centre for Cultivated Plants, Institute for Grapevine Breeding Geilweilerhof, Siebeldingen, Germany

**Keywords:** *Vitis vinifera* L., grapevine breeding, climate change, QTL analysis, abiotic stress, sunburn browning and necrosis, cool climate viticulture, MAS (marker assisted selection)

## Abstract

Grape sunburn is an abiotic stress response induced under heat wave conditions. Heat stress is reaching new dimensions in terms of intensity and frequency in European cool-climate wine-growing regions. The damage to grape berries manifests in browning and shriveling, leading to yield loss and can reduce wine quality. Established management strategies like defoliation of the cluster zone in order to reduce fungal infection pressure could enhance this problem. Climate-adapted cultivars that are resilient to sunburn would resolve those trade-offs in vineyard management. In recent years, grapes grown in the Palatinate wine region of Germany have been affected by sunburn at an unprecedented rate. The intensity of sunburn damage in experimental fields located in this region was assessed for five years, taking advantage of the unexpectedly frequent heat waves in 2019, 2020, and 2022. Phenotyping of the grape sunburn symptoms was carried out in a segregating F1 mapping population of ‘Calardis Musqué’ x ‘Villard Blanc’ and a number of varieties. The population consists of 150 genotypes cultivated in two adjacent plots with four plants per F1-individual each, providing sufficient grape material for a reliable evaluation. Composite interval mapping (CIM) using a genetic map and 5 years of phenotypic field data of sunburn damage revealed two strong QTLs located on the lower arm of chromosome 11 with LOD_max_ values of up to 16.3 and 26.1% of explained phenotypic variance and on chromosome 10 with a LOD_max_ value of 10.3 and 14.1% of explained phenotypic variance. The highest sunburn resilience of berries was observed based on an additive effect of a specific allelic combination within both loci. QTL regions were screened for annotated and expressed genes in developing grape berries to provide a first insight into understanding possible principles of sunburn resilience. Some current fungus-resistant varieties (PIWIs), such as ‘Calardis Blanc’, have demonstrated resilience to sunburn. The reported QTLs open new possibilities to breed for grape sunburn resilient vines using marker-assisted selection (MAS), but also the challenges are discussed here. This knowledge could facilitate the planting of vineyards with fungus-resistant, sunburn-protected new varieties to ensure yield and wine quality while making viticulture more sustainable.

## 1 Introduction

Climate change is expected to progress even further worldwide in the coming decades, resulting in unprecedented weather extremes that will challenge the economic viability of agricultural production in every aspect (van Leeuwen et al., 2024). In the last couple of years, weather conditions in Germany have varied considerably between very humid and warm seasons resulting in downy mildew epidemics and hot, dry summers, with alcohol rich vintages, untypical aromas and wine stylistics (Töpfer and Trapp, 2022). The cultivation of traditional grapevine varieties is under increasing pressure due to the loss of fungicidal agents to combat biotic stresses, while at the same time recurrent heat waves and other abiotic stresses result in the loss of yield and typical wine styles in originally cool climate regions. New varieties that are better adapted to abiotic stress induced by the expected change of weather conditions could ensure future production in these areas and contribute to the continued existence of this unique cultural landscape, where wine and tourism are often very important economic factors (Tafel and Szolnoki, 2020).

So far, sunburn resilience of grapes played a minor role in grapevine breeding for cultivars adapted to cool climate. Due to the effects of climate change, in recent years unusual heat waves occurred frequently causing massive sunburn damage in German viticulture (Gambetta et al., 2020). Sunburn is one of the common types of berry shrivel disorders and occurs on fruits exposed to direct sunlight, especially in the heat of the afternoon (Krasnow et al., 2010; Bondada and Keller, 2012; Keller et al., 2016). The berry symptoms start with browning of the epidermis and ranges over necrotic spots to complete berry desiccation. Those are reported to be the results of an combination of excessive photosynthetically active radiation (PAR) and UV radiation as well as high temperature, that can be exacerbated by other stress factors such as water deficit (Gambetta et al., 2020). Gambetta et al., 2020 summarize several damage levels of grape sunburn: (1) degradation of waxes resulting in dehydration, (2) destruction of chlorophyll and cell compartmentalization with subsequent oxidation of polyphenols and browning, (3) cell death in the epidermal layers and exocarp as evidenced by a higher electrical conductivity in berry skin. Grape sunburn damage causes significant yield and quality losses with reduced market value as reviewed in detail by Gambetta et al., 2020. This findings were recently confirmed by reports of a decrease in must yield of up to 30% and that resulting wines are more yellow colored with oxidative characters (Rustioni et al., 2023; Szmania et al., 2023). Next to the reconsideration of viticultural practices, like canopy management and trellis systems, the development of new grapevine cultivars with improved resilience to sunburn damage is advised, but currently there is a lack of knowledge for an efficient selection in grapevine breeding (Bondada and Keller, 2012; Keller et al., 2016; Delrot et al., 2020). Irrespective of this, significant differences in the resilience against sunburn have already been observed between grapevine cultivars (Müller-Thurgau, 1883; Krasnow et al., 2010; Rustioni et al., 2015). This implies genetically determined parameters identifiable in a classical Quantitative Trait Locus (QTL) analysis based on a F1-crossing population segregating for the trait under examination. Our approach has led to the first QTLs for sunburn resilience in grapevine berries and opens up a perspective for application in marker-assisted selection (MAS). Thus, the work contributes to the increase of breeding efficiency for improved grapevine varieties.

## 2 Materials and methods

### 2.1 Plant material and phenotypic evaluation

**Figure 1:**
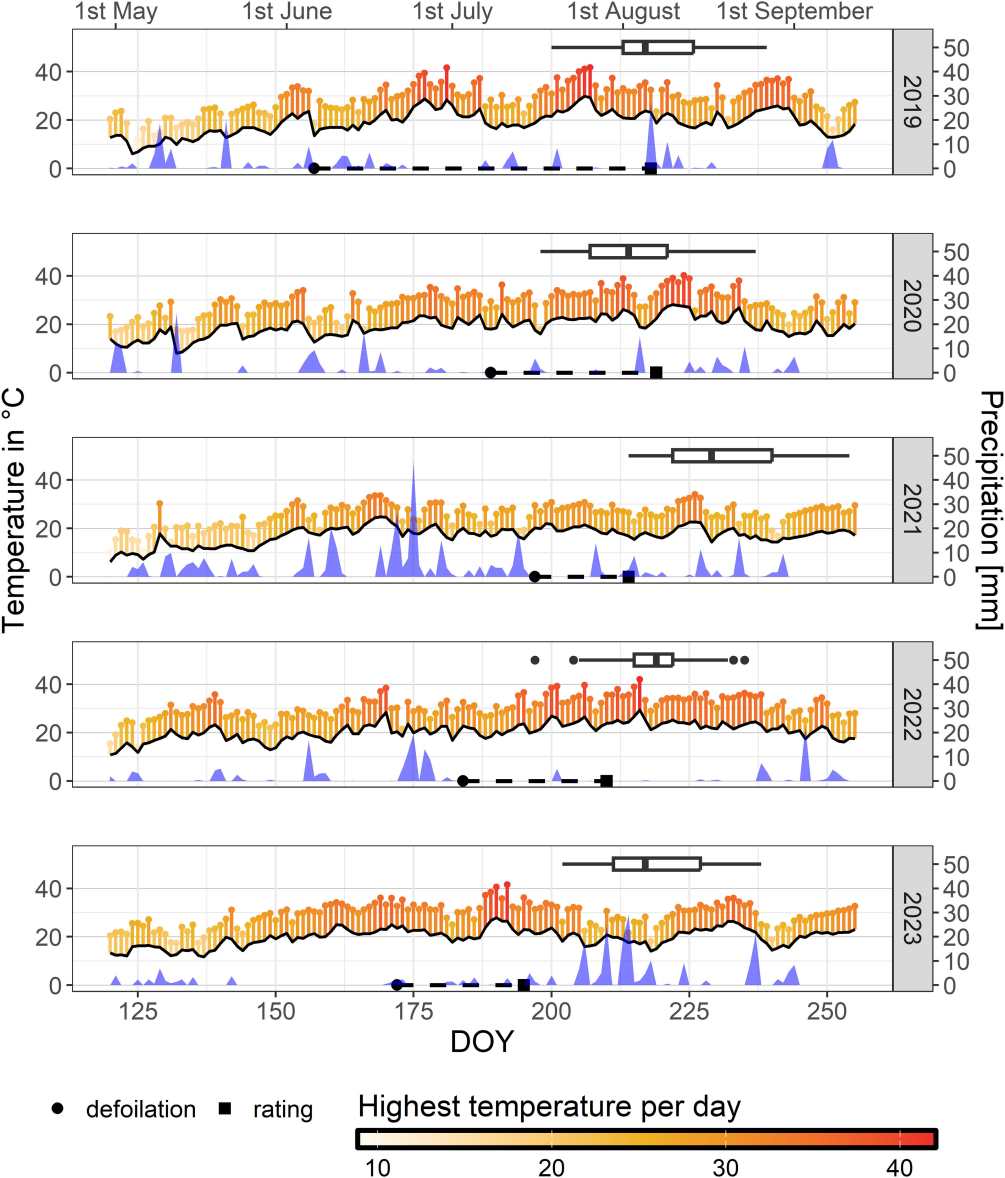
Maximum air temperature values (pinheads), mean daytime temperature (black line) 20 cm above ground level and sum of precipitation measured at the weather station “Siebeldingen (AGM 088)” in 150 m distance to the experimental plot for each day of the seasons 2019 to 2023. Dates of defoliation (circle), rating of sunburn damage (square), and dates of veraison (box plot) within the examined mapping population CMxVB are indicated for each year.

A biparental F1 mapping population of ‘Calardis Musqué’ (*V*IVC-No. 4549) x ‘Villard Blanc’ (*V*IVC-No. 13081) (abbreviated CMxVB) with 150 genotypes was used for the investigation of grape sunburn resilience. The experimental vineyard was established at JKI Geilweilerhof, Siebeldingen, Germany (49°1254”N 8°0248”E) and consists of two plots, Gf-1 and Gf-2, consecutively planted as exact copies with four vines of each genotype within one vineyard grafted on rootstocks of Selection Oppenheim 4 (SO4, *V*IVC 11473). The vines were planted in 2010 in a vertical shoot positioning (VSP, south north oriented) trellis system and a plant density of 5,000 vines per hectare (2 x 1 m spacing). Pruning is carried out on a flat arch with approx. 10–12 buds per shoot. Parental varieties were co-cultivated in the same plots. Foliage pruning was done 3 weeks after flowering in each season. Defoliation in the cluster zone was carried out later (date indicated in **Figure 1**) manually on the eastern side of the canopy, in order to achieve better aeration reducing the risk of mildew and *Botrytis* infections. Sunburn evaluation was conducted in the years 2019 – 2023, when damage became obvious. Additionally, after the heat event in 2019, sunburn assessment data were collected for 83 cultivars from two sets (international and national important cultivars) of the grapevine collection at JKI Geilweilerhof (**Table 1**). The used weather data was recorded by the station “Siebeldingen (AGM 088)” operated by the Agrarmeteorologie RLP (https://www.wetter.rlp.de/), located 150 m from the experimental plots (**Figure 1**).

**Table 1:** Phenotypic evaluation of sunburn damage in the germplasm repository of JKI Geilweilerhof (DEU098) under field conditions in the year 2019, ranging from 1 = no damage to 9 = massive necrotic damage.

INTERNATIONAL collection	*V*IVC number	Sunburn	NATIONAL collection	*V*IVC number	Sunburn	Other plots at JKI	*V*IVC number	Sunburn
ALIGOTE	312	2	CABERNET MITOS	15499	1	AIREN	157	2
CINSAUT	2672	2	CHARDONNAY BLANC MUSQUE	2456	1	CALANDRO	21797	6
PALOMINO FINO	8888	2	GEWUERZTRAMINER	12609	1	CALARDIS BLANC	22828	2
TEMPRANILLO TINTO	12350	2	SCHEUREBE	10818	1	CALARDIS MUSQUÉ	4549	7
TREBBIANO TOSCANO	12628	2	SILVANER GRUEN	11805	1	FELICIA	20348	2
WELSCHRIESLING	13217	2	DOMINA	3632	2	FELICIA MINIMAL PRUNING	20348	1
ALVARINHO	15689	3	KERNER	6123	2	GF.2004-043-0010	NA	2
ASSYRTIKO	726	3	MUSKATELLER GELB	41578	2	GF.2010-011-0048	NA	3
BARBERA NERA	974	3	OPTIMA	8791	2	PHOENIX	9224	7
CABERNET FRANC	1927	3	RIESLANER	10073	2	REBERGER	19999	3
CHARDONNAY BLANC	2455	3	GEWUERZTRAMINER	12609	2	REBERGER (MINIMAL PRUNING)	19999	2
CLAIRETTE BLANCHE	2695	3	AUXERROIS	792	3	SEYVE VILLARD 39-639	11670	6
GARNACHA TINTA	4461	3	CABERNET DORSA	20002	3	SEYVE VILLARD 39-639	11670	5
TANNAT	12257	3	MUELLER THURGAU WEISS	8141	3	VILLARIS	20347	1
TOURIGA NACIONAL	12594	3	REGENT	4572	3	VILLARIS (MINIMAL PRUNING)	20347	1
VELTLINER GRUEN	12930	3	SOLARIS	20340	3	HEUNISCH WEISS	5374	6
ARAMON NOIR	544	4	PORTUGIESER BLAUER	9620	4			
CABERNET SAUVIGNON	1929	4	BLAUFRAENKISCH	1459	4			
CARMENERE	2109	4	CHARDONNAY BLANC	2455	4			
MERLOT NOIR	7657	4	CHASSELAS BLANC	2473	4			
MUSCAT A PETITS GRAINS BLANCS	8193	4	MORIO MUSKAT	7996	4			
PRIMITIVO	9703	4	RIESLING WEISS	10077	4			
RKATSITELI	10116	4	ACOLON	17123	5			
SAUVIGNON BLANC	10790	4	DAKAPO	14728	5			
SEMILLON	11480	4	ELBLING WEISS	3865	5			
MONASTRELL	7915	5	FABERREBE	4029	5			
RIESLING WEISS	10077	5	PINOT NOIR	9279	5			
ROUSSANNE	10258	5	SAINT LAURENT	10470	5			
SYRAH	11748	5	PINOT BLANC	9272	6			
VIOGNIER	13106	5	PINOT GRIS	9275	6			
CHENIN BLANC	2527	6	PINOT MEUNIER	9278	6			
PLAVAC MALI CRNI	9549	6	PINOT PRECOCE NOIR	9280	6			
CARIGNAN NOIR	2098	7	DORNFELDER	3659	7			
COLOMBARD	2771	7	DUNKELFELDER	3724	7			
GAMAY NOIR	4377	7	SCHIAVA GROSSA	10823	7			
PINOT NOIR	9279	7	BACCHUS WEISS	851	8			
DOLCETTO	3626	8						
NEBBIOLO	8417	8						

**Figure 2:**
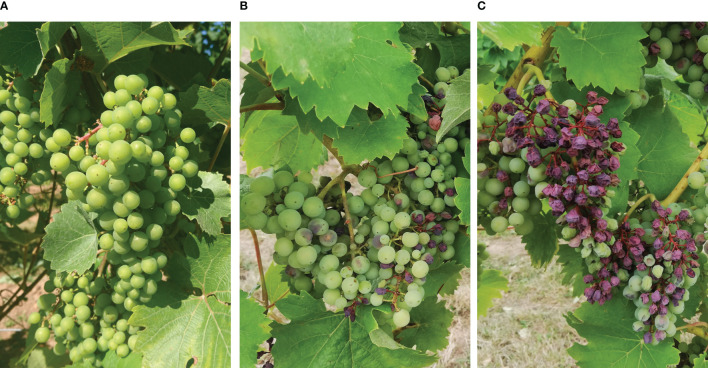
Sunburn damage (brown berries) on grapes. The plants show different sensitivity: left – 1=no sunburn damage visible **(A)**; middle - 5=medium damage with necrotic sunburn defects of a smaller number of berries **(B)**, right - 9=massive necrotic damage with high yield losses in exposed bunches **(C)**.

Sunburn damage on berries was phenotyped in the field using a 5-class rating system according to the International Organisation of Vine and Wine (OIV) descriptor 404 for “thermal stress” (1=very low; 3=low; 5=medium; 7=high; 9=very high) (OIV, 2024). For the assessment the rating scheme was modified as followed: 1=no sunburn damage visible; 3=few berries with discoloration and local necrotic sunburn; 5=medium damage with necrotic sunburn defects of a smaller number of berries, 7=a higher number of necrotic damaged berries causing minor yield losses; 9=massive necrotic damage with high yield losses in exposed bunches (**Figure 2**). One class rating was evaluated for the 4 individuals overall per plot. The data was recorded using the PhenoApp (Röckel et al., 2022).

### 2.2 Development of locus specific markers

The used SSR marker map is a revised version of the one published by Zyprian et al., 2016. SSR markers were re-analysed to address gaps and uncertainties, and additional markers were included. For fine mapping, primers were deduced based on the grapevine reference genome PN40024 T2T (v5) (Shi et al., 2023) to create locus specific SSR markers on the lower arm of chromosome 11 and the upper arm of chromosome 10. This was done using WebSat (Martins et al., 2009) and CLC Genomics Workbench (Qiagen, Hilden, Germany).

### 2.3 Candidate gene identification

The QTL regions, defined by the SSR marker positions flanking the LOD_max_^-1^ interval on the PN40024 v5 reference genome, were investigated on sequence level for putative functional genes using Blast2GO provided by Grapedia (grapedia.org, 2024). In addition, positions of these markers in PN40024 v4.2 (Velt et al., 2023) were extracted, to explore the GRape Expression ATlas (Velt, in press) for relevant gene expression studies in relevant organs and within the region of interest. For the v5 version of the reference genome, no studies have been submitted to this database so far. Hence, the v4.1 (Blast2GO) annotation file downloaded from Grapedia was included (**Supplementary Table 1**). For the screening of the expression studies, only experiments with no treatments and control plants were considered. All cultivars with reports for berry samples within the general developmental stages “Green berries” and “Ripening berries” and extracted organ as “Berries” were included. The data for the extracted QTL regions containing the results of the expression studies are provided in **Supplementary Table 1**. Subsequently, the genes of interest were compared between v5.1 and v4.1, with consideration given to their activity and putative function. The OneGene platform (https://onegene-causality-weaver.disi.unitn.it/vitis/network/) was used to perform network analysis (Pilati et al., 2021) to identify correlating genes.

### 2.4 Statistical analysis

All analyses were performed via R Statistical Software v4.2.2 (R Core Team, 2022) in *RStudio* v2024.4.0.735 (Posit team, 2024). Best-linear-unbiased-predictors (BLUPs) (Piepho et al., 2008) for all years were determined with the R-package *phenotype* (Peng, 2020) and the genomic heritability (*h^2^*) was calculated with a simple kinship-matrix from *r/QTL* (Broman et al., 2003) and the package *heritability* (Kruijer et al., 2014). Pearson correlation coefficients with corresponding *p*-values were computed with the R core package *stats*. The genetic map was constructed with *OneMap* (Margarido et al., 2007). Linkage groups were numbered according to the chromosomes of the reference genome. Interval mapping and composite interval mapping in the QTL analysis were performed with *fullsibQTL* (Gazaffi et al., 2014). Cofactors were selected via multiple linear regression function from the *fullsibQTL* package. Interactions between the two QTLs were calculated with *r/QTL*. Additional plots were designed with *ggplot2* (Wickham, 2016).

## 3 Results

### 3.1 Examination of heat wave events, viticultural management and date of ratings

Field evaluations were conducted for five consecutive years starting in 2019 to assess a mapping-population for sunburn sensitivity or resilience, respectively. Sunburn events after heat incidents were observable in varying degrees in 2019, 2020, 2022 and were rather weakly expressed in 2021 and 2023. The temperature conditions with mean and maximum temperature per day over the growing seasons within the years of study are shown in **Figure 1**.

**Figure 3:**
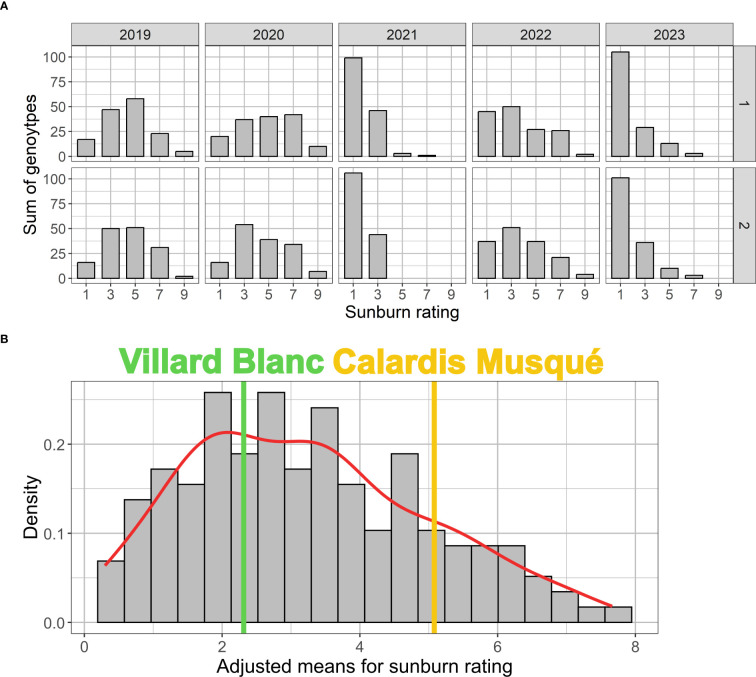
Histograms with independent sunburn damage rating in both plots (Gf-1 and Gf-2) of the ‘Calardis Musqué’ x ‘Villard Blanc’ F1-population. **(A)** Distribution for the five seasons investigated. The rating ranges from “no visible sunburn damage” (=1) to “massive necrotic damage” (=9). The histogram **(B)** shows the BLUP-adjusted mean for all genotypes over the five years, the corresponding kernel density estimate (red), and the adjusted means for the parental cultivars ‘Calardis Musqué’ (yellow) and ‘Villard Blanc’ (green).

The study was initiated following observations of massive sunburn damaged grapes in early August 2019 and the obvious segregation between the genotypes of the CMxVB population. The conditions in the five years of the study were as follows:

Year 2019: Defoliation of the cluster zone was carried out manually in early June (6^th^) according to viticultural practices. A natural heat peak at the end of June with a maximum of 41.6°C on June 30th and a second heat peak exceeding 40°C over three days from 24th to 26th July with a maximum of 41.7°C caused massive sunburn damage at single berries or partly whole bunches. The extent of damage per genotype was rated separately on August 6th for the two repetitive plots at JKI Geilweilerhof (**Figure 3**). Differences in the degree of damage between the F1-genotypes were evident and ranged from visually unaffected berries to massive necrotic sunburn symptoms with substantial yield losses.
Year 2020: A deliberately late defoliation on July 7th – 8th in 2020 intended to increase the risk of sunburn, combined with a seven-day heat period at the end of July with temperatures above 32°C and a peak temperature of 38.9°C on July 31, was sufficient to induce sunburn symptoms in sensitive F1-individuals. Segregation within the population was observed (**Figure 3**) as documented on August 6th 2020. A second heat event around August 11th occurred after the rating and was not considered in the study.
Year 2021: Defoliation was done on July 16th. No similar high temperatures as in the previous two years were reached in 2021. Maximum temperature occurring during the season was 34.1°C. Therefore, only minor sunburn damage were observed in highly sensitive F1-genotypes. Rating was done at August 2nd (plot Gf-1) and 5th (plot Gf-2).
Year 2022: The first half (Gf-1) of the experimental vineyard was defoliated on July 3rd and 4th at BBCH 73-75, while the second half (Gf-2) was defoliated between July 11th and 14th at BBCH 77-79. An initial heat event occurred in June with temperatures up to 38.4°C, followed by a warm and dry period in the second half of July with temperatures reaching 39.6°C. This episode caused significant damage to sensitive F1-individuals assessed on July 29th. A third heat event around August 4th with temperatures of up to 42°C occurred after the evaluation and is not taken into account.
Year 2023: Defoliation on June 21st and weather conditions with five days above 35°C from July 7th to July 11th and a maximum of 41.6°C on the last day were not sufficient to induce severe sunburn damage. This resulted in a less distinct segregation within the F1 population as evaluated on July 14th and shown in **Figure 3**.

### 3.2 Sunburn damage effects

Extend of sunburn damage within the CMxVB population for the individual years of investigation is shown in the histograms given in **Figure 3A**. By visual examination of the histogram (**Figure 3B**) and the QQ-plot (**Supplementary Table 1**) based on the BLUP adjusted mean values for the population over all the years, an approximate normal distribution can be assumed, even though this was not confirmed by the Shapiro Wilk test (p-value: 0.001692). The comparison of variation within the F1-population with the observed sunburn resilience of the parents reveals a transgressive segregation, indicating multiple loci to be involved in trait expression.

### 3.3 Correlation between sunburn damage and the developmental stage of the grapes

The individuals of the CMxVB population segregate strongly over a period of about 6 weeks for their time of veraison (Frenzke et al., 2024), which is the onset of berry ripening. This means that the grapes of individual genotypes are at different stages of berry development at the time of heat exposure. Therefore, the impact of the developmental stage on the sunburn damage was checked with a Pearson correlation analysis. The day of the veraison combined with the sunburn rating resulted in rather weak correlation of -0.14 and non-significant p-value = 0.08. This suggests a subordinate influence of the veraison itself on the degree of damage in this population.

### 3.4 QTL analysis of sunburn sensitivity

QTL analyses were performed by using their BLUP value calculated with the rating of four plants over both plots and all five years. Detailed results are presented in **Supplementary Table 2**. The calculated genomic heritability ranges from 0.29 to 0.65 between the years due to the different characteristics of the heat stress events. The overall BLUP with 0.59 *h^2^* suggests a strong genetic influence on sunburn resilience.

**Figure 4:**
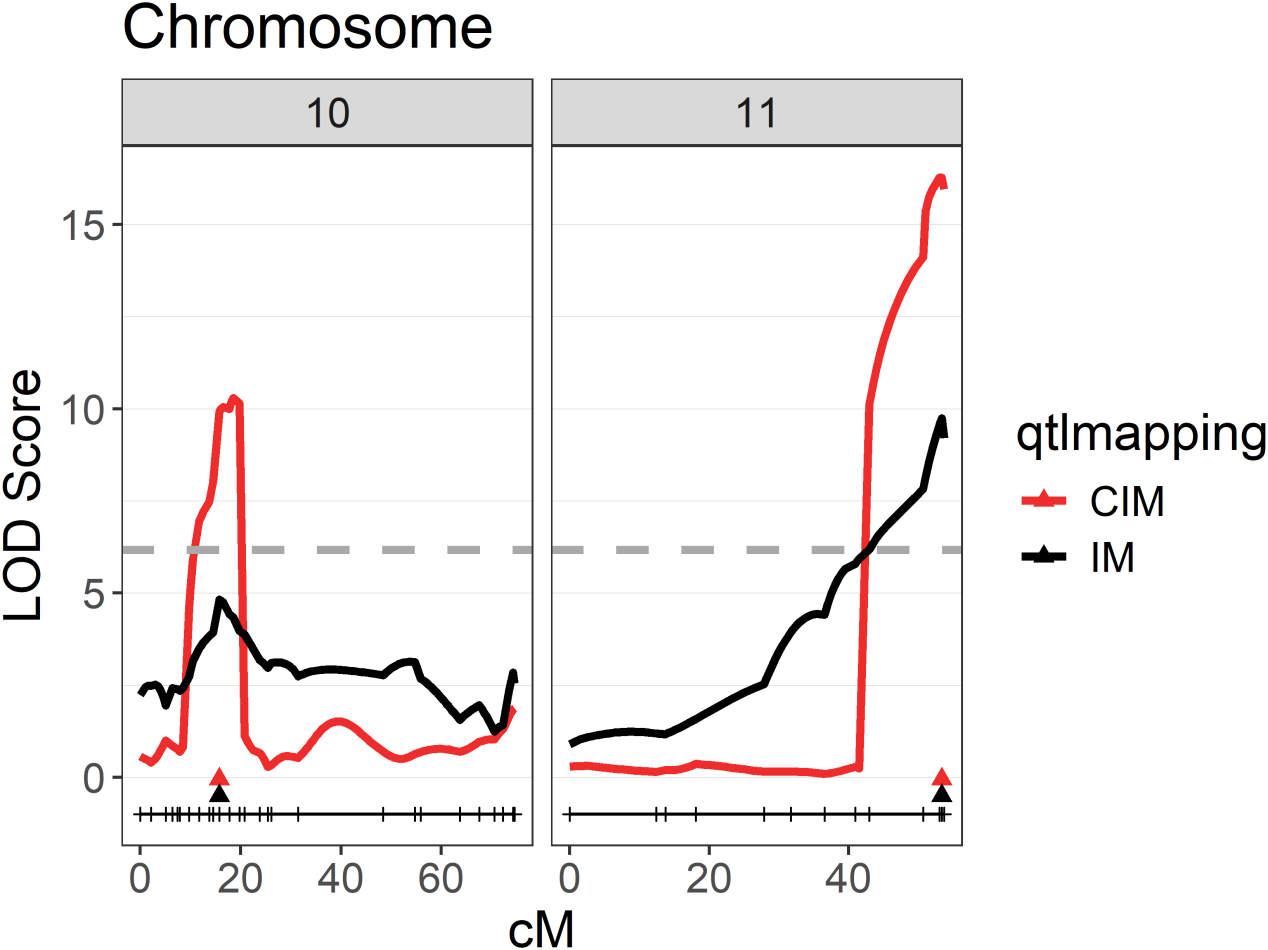
QTLs for sunburn resilience on chromosomes 10 and 11 of the mapping population ‘Calardis Musqué’ x ‘Villard Blanc’. QTLs calculated based on the BLUP-values for sunburn damage observed for each F1-genotype in five seasons on two plots from 2019 to 2023. The genome-wide 5% significance threshold for the composite interval mapping with a LOD value of 6.17 is indicated as a dashed line.

When performing interval mapping (IM) using the BLUP values, a first QTL on chromosome 11 with a maximum logarithm of the odds (LOD_max_) of 9.8 and a second QTL on chromosome 10 with a LOD_max_ of 4.8 were identified (**Figure 4**). Those QTLs were further confirmed by composite interval mapping (CIM), resulting in a peak on chromosome 11 with a LOD_max_ of 16.3 and one on chromosome 10 with a LOD_max_ of 10.3. The first QTL resulted in 26.1% and the second QTL in 14.1% explained phenotypic variance.

### 3.5 Construction of the revised genetic map with additional markers

**Figure 5:**
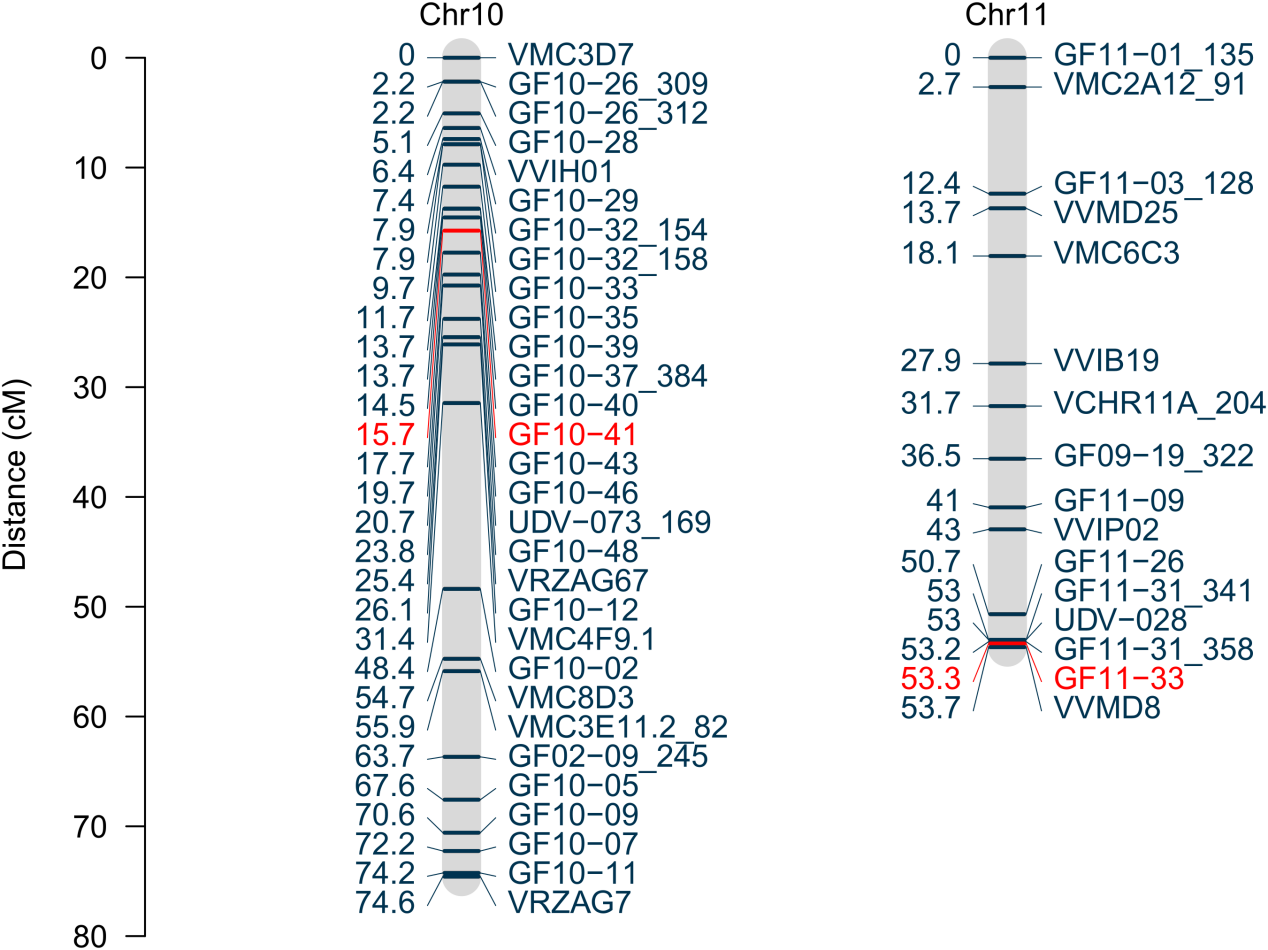
Linkage groups representing the chromosomes 10 and 11 based on SSR markers. Genetic positions are displayed in centiMorgan with the according marker names. The LOD peak markers for each QTL are marked in red.

To increase the resolution of the genetic map within the two identified chromosomal regions, 28 SSR-markers were newly developed. Fifteen of those markers segregated in the mapping population and were integrated into the map. A further fine mapping of the lower arm of chromosome 11 based on the given 150 genotypes is restricted due to the lack of recombination events in this region. This resulted in 13 added markers on chromosome 10 and three newly mapped makers on chromosome 11 (**Figure 5**) compared to the previously published map (Zyprian et al., 2016). Marker positions and primer sequences are attached in **Supplementary Table 2**. The revised SSR marker map consists of 392 SSR markers on 19 linkage groups with a total length of 1401.5 cM. The average *R* ^2^ of the coverage to the grapevine reference genome PN40024 v5sequence is 94.4% for chromosome 10 and 84.5% for chromosome 11.

### 3.6 Investigation of the genetic interaction between the two loci

**Figure 6:**
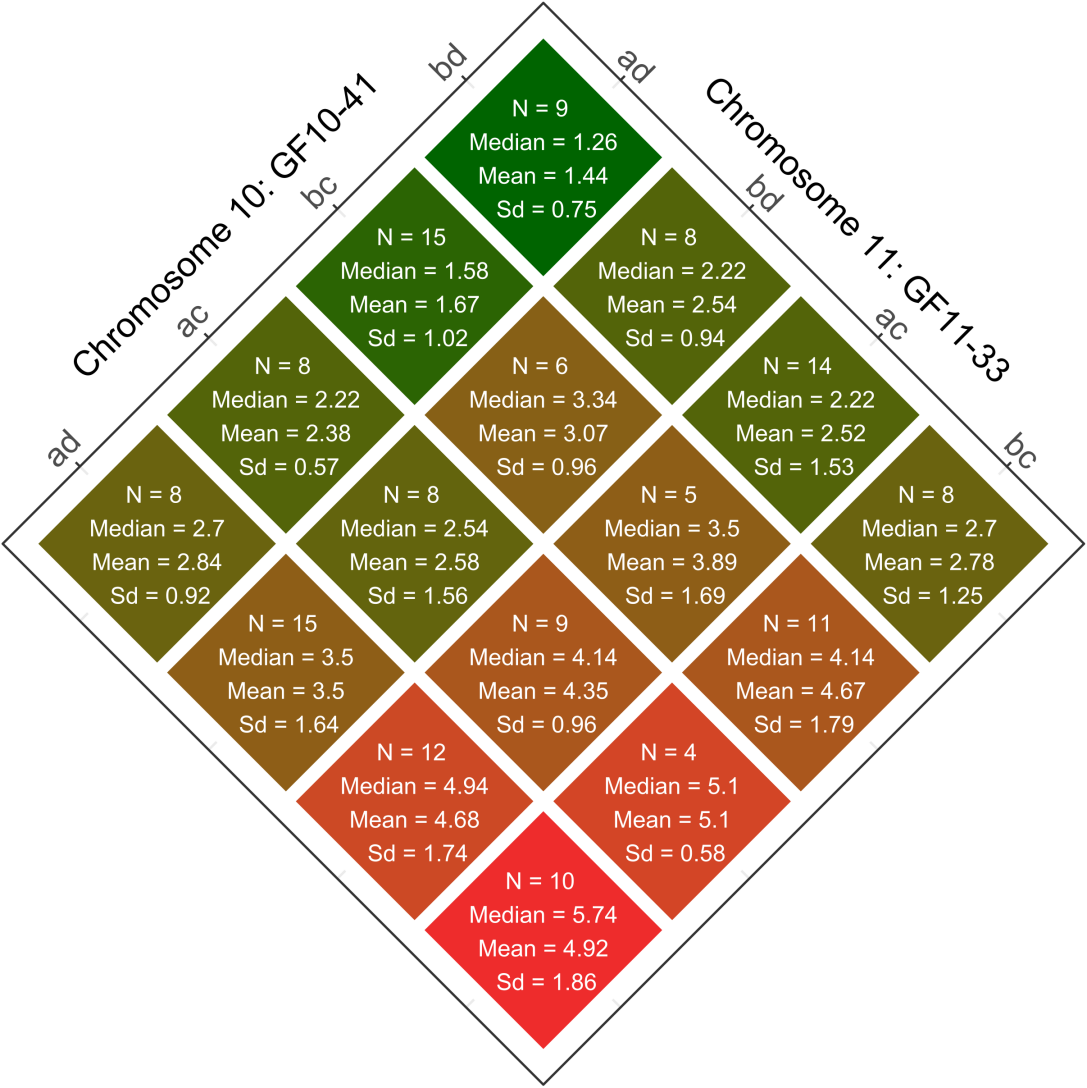
The interaction plot shows the observed phenotypic effects of the allelic combinations on sunburn resilience given by the QTL linked SSR markers on chromosomes 10 (GF10-41) and 11 (GF11-33) in the F1-Individuals of the population CMxVB. Coloration and order depend on the median values of sunburn damaged grapes from low (top/green) to high (below/red). Also shown are the number of F1-individuals (N), phenotypic median, means and standard deviation (Sd) for the individual groups.

Two of the newly developed, integrated, and fully informative SSR markers are located in the respective centres of the two QTLs. They show the highest LOD values and were therefore selected to study the phenotypic effects. The strength of the mediated sunburn resilience varies depending on the allelic combination within both QTLs (**Figure 6**). The 16 allele combinations resulting of these two markers, namely GF11-33 (PN40024_v5: chr11, 19,740,009bp) on chromosome 11 and GF10-41 (PN40024_v5: chr10, 5,242,306bp) on chromosome 10 with two distinct alleles each are demonstrated. The interaction plot shows the identified allelic combinations for lowest (Chr10:bd/Chr11:ad) and highest (Chr10:ad/Chr11:bc) sunburn damage as well as their intermediate stages (**Figure 6**).

In the QTL on chromosome 11 (represented by GF11-33) the allele d, derived from ‘Subereux’ (*V*IVC-No. 12031) a parent of ‘Villard Blanc’, is associated with sunburn resilience, especially when paired with allele a originating from ‘Bacchus Weiss’ (*V*IVC-No. 851), a parent of ‘Calardis Musqué’. In the second QTL on chromosome 10 (represented by GF10-41), the allele b, inherited from ‘Seyval Blanc’ (*V*IVC-No.11558) the second parent of ‘Calardis Musqué’, mediates improved resilience.

### 3.7 Potential candidate genes for sunburn resilience

The sequence of the reference genome underlying the QTL regions on chromosomes 10 and 11 were investigated for potential candidate genes that may influence sunburn resilience. In PN40024 v5 genome, the flanking markers of the QTL on chromosome 10 (GF10–40 at 4.769 Mb and GF10–46 at 5.650 Mb) cover a region of 880 Mb that includes 108 annotated genes, whereof 97 have reported putative functions. The QTL on chromosome 11 spans a slightly larger region of 1,687 Mb (between the flanking markers GF11–26 at 18.206 Mb and VVMD8 at 19.893 Mb) and includes 179 annotated genes. 141 of them have reported putative functions (**Supplementary Table 1**).

**Figure 7:**
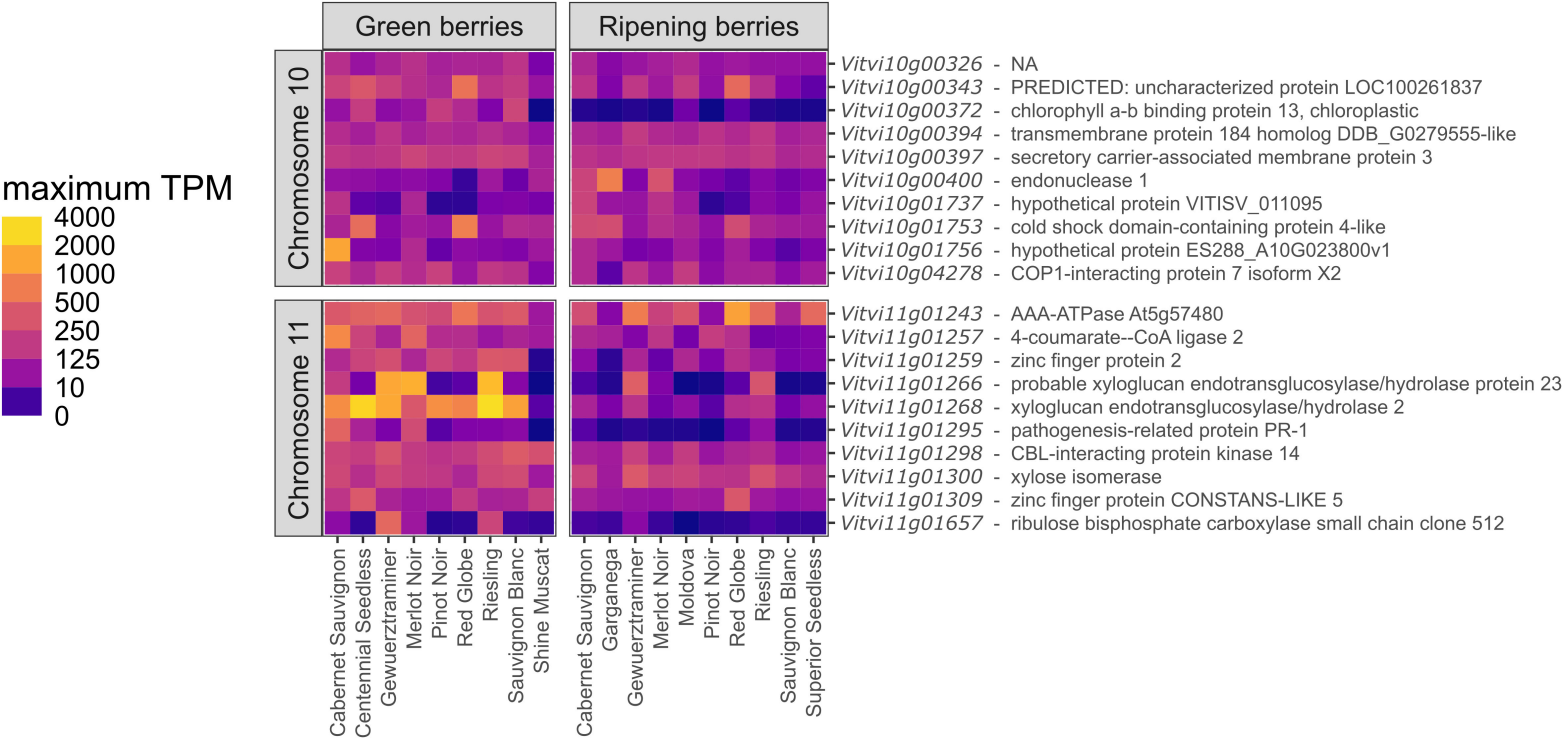
The heat map shows a comparison of gene expression levels in transcripts per million (TPM) between different cultivars in the developmental stages “Green berries” and “Ripening berries”. The 10 highest expressed genes per QTL region on chromosome 10 and 11 are displayed, summarized through the maximum expression rate per cultivar in one experiments at the same developmental stage. Next to the GeneID, the putative functions with the best hit reported in PN40024 v4.1 are listed.

Due to missing expression studies referring to the PN40024 v5 genome so far, the QTL regions were additionally transferred via the previously mentioned flanking markers to the reference genome version PN40024 v4.2 (40X). The extracted sequence regions were used to explore the available expression studies (**Figure 7**). On chromosome 10, the confidence interval LOD_max_^-1^ spans a sequence of 778 Mb (between 4.339 Mb and 5.117 Mb) including 91 annotated genes with reports of 60 putative functions. On chromosome 11, the interval covers the region between 18.102 Mb and 19.775 Mb with a total length of 1.672 Mb, including 138 annotated genes containing 83 with putative functions (**Supplementary Table 1**). Therefore, an increase of sequence length as well as of annotated genes was observed in the recent reference genome.

To focus on annotations with the highest candidate gene potential, the expression levels in green and ripening berries based on the available data of 13 bioprojects including 12 cultivars were investigated using the GREAT database. The top ten genes in terms of highest measured expression level in one experiment for both developmental stages (green berries and ripening berries) in a cultivar are presented in **Figure 7**. The widest range in transcripts per million (TPM) between two cultivars at the same stage is 3037 in ‘Riesling’ compared to 6 TPM in ‘Shine Muscat’ for *Vitvi11g01268*. While some annotated genes seem to be more or less equally expressed (e.g. *Vitvi10g00372*, *Vitvi10g00397*, *and Vitvi11g01300*) between the cultivars at the same stage, others show a strong variance (e.g. *Vitvi11g01266*, and *Vitvi11g01268*).

### 3.8 Sunburn damage observed for relevant cultivars

Sunburn symptoms were recorded for two parts of the grapevine collection (national cultivars and international cultivars) at JKI Geilweilerhof and for some selected varieties and breeding lines subsequently to the heat wave of 2019. Under the local weather conditions, as described earlier, those cultivars reacted very differently considering the observed sunburn damage. Very sensitive cultivars like ‘Dunkelfelder’ (*V*IVC-No. 3724), ‘Dornfelder’ (*V*IVC-No. 3659), ‘Schiava Grossa’ (*V*IVC-No. 10823), and ‘Bacchus Weiss’ (*V*IVC-No. 851) were found, as well as resilient ones like ‘Calardis Blanc’ (*V*IVC-No. 22828), ‘Silvaner Gruen’ (*V*IVC-No. 11805), and ‘Tempranillo Tinto’ (*V*IVC-No. 12350).

## 4 Discussion

### 4.1 Sunburn damage due to heat waves in an historical context

With ongoing climate change, the incidence of grapes damaged by sunburn is increasing. However, grape sunburn events are not an entirely new phenomenon for the German wine-growing regions, as Müller-Thurgau already reported in 1883. At that time, viticulturists had already correctly identified the symptoms of sunburn damage on berries. Müller-Thurgau indicated years, in which moist and cold weather conditions were suddenly followed by hot sunny days as a particular risk for sunburn on unshaded grapes. Gambetta et al., 2020 report sunburn incidents in German vineyards in 1892, 1930, 1947, 1966, 1973, 1998, 2007, 2012, and 2019. In addition, the years of 2020 and 2022 continued with sunburn events as reported in the present and further studies (Friedel and Müller, 2022; Waber et al., 2023). Accordingly, in Germany’s second largest wine-growing region Palatinate, grapes were affected by unusually high temperature events resulting in remarkable sunburn symptoms in three of the five years between 2019 and 2023, namely 2019, 2020 and 2022. This problem is exacerbated by the common local viticultural practice of extensive defoliation, which many winegrowers perform to improve the microclimate with sun light exposure for aroma formation, rapid drying of grape bunches for health maintenance, improved spray penetration and berry coloration (Drenjančević et al., 2018; Gambetta et al., 2020).

Müller et al., 2023 mentioned, that the likelihood of a berry developing sunburn necrotic symptoms depends on the combination of intensity and duration of the heat event, as well as other biotic and abiotic factors. In the present study sunburn damage in sensitive genotypes was obvious for the years 2019, 2020, and 2022 after hot periods, with peaks exceeding 40°C air temperature (**Figure 1**). The symptoms occurred independently of how much time passed since defoliation. In all years, plants could acclimate for at least seven days after leaf removal, which according to Müller et al., 2023 is the period required to adapt the berries to heat events. Thus, the damage occurred despite of adaption and could probably be explained by the much higher, climate change driven recent temperature profiles compared to the very early reports. While, Zschokke, 1930 observed fatal damage to grape berries in 1929 at maximum air temperatures of 32.5°C, while recent air temperature reached above 38.9°C (in 2020) or even 41.7°C (in 2019) resulting in symptomatic berries. The comparison between the historic data and today’s bears uncertainties. However, it could be concluded that the higher temperatures in combination with the duration of heat exposure no longer need additional surrounding circumstances such as windlessness, high humidity and low water status to contribute to the same extent to sunburned berries as in former times. Consequently, the sunburn risk has increased substantially.

Another interesting observation by Müller et al., 2023 is that water-stressed grapevines were less or equally sensitive to sunburn necrosis compared with fully irrigated plants. This is in accordance with the observations of Müller-Thurgau (1883) who postulated that water-deficient berries are better protected. On the other hand, he showed cooling effects by berry transpiration (Müller-Thurgau, 1883) and recent studies suggest that the individual water stress level might therefore be of relevance for the expressed sunburn damage symptoms. For example, changes in berry wax composition and transpiration as an result of water stress were reported (Dimopoulos et al., 2020). As all vines in the present study were grafted on the same rootstock variety and planted in the same vineyard, differences between the genotypes would be driven mainly by differential transpiration rates and foliage of the scion genotypes and should be topic of further investigations. Further environmental factors like wind, relative air humidity and global radiation may be additional factors to be considered, but have not shown any statistical significance on sunburn damage here (**Supplementary Table 4**). In accordance, Gianluca et al., 2025 found out that including radiation data did not improve the accuracy of their machine learning model to predict sunburn damages. A nuanced approach measuring the individual berries, clusters and canopy shading would be necessary, to provide further inside.

Despite the multifactorial stressors that can contribute to the expression of grape sunburn damage, the presented field data over 5 seasons indicate that heat waves have a particularly strong influence on the occurrence of sunburn in sensitive genotypes.

### 4.2 Influence of the berry ripening on sunburn sensitivity

Gambetta et al., 2020 found reports indicating that the developmental stage affects sunburn sensitivity. While some authors reported an increase in sunburn sensitivity during the berry development (Webb et al., 2010; Hulands et al., 2013, 2014) others found opposite effects (Müller-Thurgau, 1883; Zschokke, 1931; Müller-Stoll, 1939; Coombe, 1995; Gouot et al., 2019a, b) and it is concluded, that sunburn sensitivity is likely to peak during the veraison, with its major changes in berry-metabolism. The question of developmental dependence of sunburn resilience can be investigated in much more detail and resolution in a suitable cross population that is highly segregating for timing of veraison and berry maturity (Zyprian et al., 2018; Frenzke et al., 2024), than in individual varieties.

Based on the evaluation over five years, including three years of severe sunburn damage, on the given population of 150 genotypes, a clear statement can be made here: Due to a lack of significant correlation between veraison and sunburn damage, the impact of developmental stage is negligible compared to the genotypic effect.

This result is consistent with those of the QTL analysis of the sunburn data, which do not show any QTL for sunburn coinciding with the *Ver1* locus, previously identified as dominant factor for timing of veraison in the same mapping population on chromosome 16 (Frenzke et al., 2024; Zyprian et al., 2016). The results do not contradict the observed changes in sensitivity over the ripening period by other studies, when considering individual grape varieties (Düring and Davtyan, 2002; Greer et al., 2006; Abeysinghe et al., 2019), but show that the effect is of minor importance compared to the varietal variance.

### 4.3 QTL results and gene expression analysis

This study revealed two important QTLs for sunburn resilience on chromosomes 10 and 11 based on the phenotypic field data for sunburn damage of five years and the SSR marker based genetic map. To identify potentially relevant genes for sunburn resilience, all annotated genes within the QTL regions were checked for putative functionality, indicated by gene expression. Knowing that the publicly available RNA expression data sets are not perfectly adequate for a comprehensive analysis with missing phenotypes and divergent cultivars studied, the data was used to determine a general genetic activity within the relevant developmental stages. The occurrence of differing expression rates between the cultivars could be potentially related to physiological differences influencing sunburn characteristics. While berry temperatures above 45-50°C have been observed to induce brown and necrotic spots on berry skins, potentially due to oxidative stress (Griesser et al., 2024), no genes with putative corresponding functions were found to be located within the QTL regions. Heat shock proteins or superoxide dismutases known to be involved in heat stress responses from other species (Kotak et al., 2007) are absent. A MYB24-MYC2 complex has been reported as regulator of heat responses and to induce specialized metabolism pathways in grape berry skin (Zhang et al., 2023, 2024). MYB transcription factors in general were shown to play diverse roles in plant abiotic stress responses (Wang et al., 2021). Therefore, a special focus was set on *MYB* genes within the QTL regions and two genes with homology to *MYB36* (*Vitvi11g01283*) and *MYB4 isoform X1* (*Vitvi10g00345*) could be identified. The *MYB36* gene is reported as a critical positive regulator of cell differentiation and negative regulator of proliferation in the endodermis of *Arabidopsis* roots (Liberman et al., 2015). Its expression (together with *APX-1*) under a constitutive promoter resulted in enhanced heat tolerance at grain filling/milking stage in wheat (Firdous et al., 2024), showing its general involvement in plant heat stress reaction. In grapevine, differential expression patterns were observed in roots under differing phosphate concentrations (Gautier et al., 2020), but their potential role in sunburned berries remains open. *MYB4* is reported as key regulator in UV tolerance by regulating hydroxycinnamate esters with UV sunscreen functionality in *Arabidopsis* (Wang et al., 2021). For grapevine a function as transcriptional repressor of flavonoid structural genes is known (Pérez-Díaz et al., 2016) and could partly explain the poor color phenotype of sunburn damaged dark berried cultivars (Krasnow et al., 2010). All these findings make the MYB transcription factors a promising target for further studies to investigate different sunburn resilience phenotypes as well as the observed additive effects given by the allelic combinations of both QTLs (**Figure 6**). To check for a potential connection between the two *MYB* genes on both chromosomes, the OneGene network was used choosing the shared nodes function. A single gene (*Vitvi04g00472*) coding for a protein kinase domain-containing protein was identified to be positively correlated with both MYB genes and seems to be a part of a complex regulatory network.

On chromosome 11, a cluster of 16 genes with a putative protein function reported as xyloglucan endohydrolase and/or endo-transglycosylase (XTH) was identified and appears to be of particular interest (**Figure 7**). Xyloglucans play an important role in the cell wall structure and can change their properties like the expandability (Kamerling, 2007). Differences in the underlying activity of those genes in the relevant developmental stages could be a modulating factor. XTHs are highly expressed in green berries when relying on the predicted protein functions for *Vitvi11g01266* and *Vitvi11g01268*. Their expression reaches very high values up to 2016 and 3036 TPM, respectively, as measured in ‘Riesling Weiss’ (*V*IVC-No. 10077) (Duchene 2018, PRJEB45016; https://great.colmar.inrae.fr/). On the other hand, there are low expression rates in e.g. ‘Sauvignon Blanc’ (*V*IVC-No. 10790) and ‘Shine Muscat’ (*V*IVC-No. 22688). The very high expression intensity reported for the two *XTH* annotations is particularly noteworthy, but could partly be an artefact formed by the overall expression level of the 16-gene cluster, as expression studies can hardly distinguish very similar transcripts. Nevertheless, the observed differences in the expression patterns between cultivars could also be an explanation for varietal differences, but this theory needs to be proven by collecting reliable sunburn damage phenotypes of the cultivars studied. During the ripening stage, the expression level of both reported genes decreases in all varieties.

Interestingly, ‘Shine Muscat’ is a table grape with a bright yellow-green pericarp and was reported to be very sensitive for skin browning and therefore grape production includes bagging of bunches to prevent reduced market value. On initial observations, the browning phenotype (called “*Kasuri-shou*”) appearsto be visually similar to sunburn browning symptoms (Katayama-Ikegami et al., 2017). Expression studies identified a specific upregulation of a polyphenol oxidase (*VvPPO2*) and two synthase genes to be associated with berry skin browning (Suehiro et al., 2014). While *VvPPO2* location was uncertain in the study of Suehiro et al., 2014, a Blast analysis of the reported primer sequences on v5 of the reference genome in the present study revealed the position of *VvPPO2* on chromosome 10 at 5.661 Mb, only 11 kb downstream of the lower marker flanking the sunburn QTL. In addition, the primer binding sites of the paralog *VvPPO1* are also located in this region on two positions (5.682 and 5.699 Mb), but this gene doesn’t seem to be linked to browning (Suehiro et al., 2014). As silencing of a *PPO* gene resulted in necrotic lesion on walnut leaves (Araji et al., 2014). This knowledge makes *VvPPO2* an additional potential candidate gene to be involved in grape sunburn response. The position slightly outside the LOD_max_^-1^ confidence interval could be explained by the statistical properties of the QTL analysis. Upstream of the QTL on chromosome 10, a SNP slightly exceeding the significance threshold for sunburned leafs was identified in a GWAS analysis based on 279 grapevine cultivars. The SNP was associated with an 15.4 kDa class V heat-shock-protein (Coupel-Ledru et al., 2024) and the underlying gene could serve as a further possible candidate.

Next to responsive reactions, the sunburn resilience mechanism could be based also on a preemptive measure, which shifts the focus back to the XTHs. Xyloglucan polysaccharides are the main hemicellulose group of the primary cell walls in dicotyledonous plants and can comprise up to 20% of the wall dry matter. Xyloglucans play an important role in interlacing the cellulose microfibrils and have been strongly implicated in the regulation of cell wall extension, particularly in conjunction with the enzyme XTH (Kamerling, 2007). A heat stress comparison between seedlings of the two grapevine varieties ‘Shenfeng’ (*V*IVC-No. 24745) and ‘Shenhua’ (*V*IVC-No. 24058) showed a significant higher expression level of XTH genes after a 45 °C heat treatment for 3 and 6 h for the thermo-tolerant variety Shenhua (Zha et al., 2020). Another experiment investigated four sunlight exposure strategies for bunches in viticulture: (1) basal leaf removal at green berry stage, (2) half-leaf and (3) full leaf removal at veraison as well as (4) leaf moving at veraison. The different leaf management strategies all resulted in an up regulation of four XTH encoding genes in not quite ripe berries (He et al., 2020). Overall, these results show that the XTH genes are linked to abiotic stress response, especially heat and light, making the identified gene cluster an interesting candidate to explain improved sunburn resilience. Other preemptive modifications of the cell wall have already been identified to be important, for example epicuticular waxes. As a coating for grape berries, the wax layer was identified to effectively limit sunburn browning in ten white grape varieties (Domanda et al., 2024). Wax layers were found to differ in ultrastructure between varieties and a QTL for impedance of berries as an indirect measure for the assessment of cuticle thickness and permeability was identified on chromosome 11 (Herzog et al., 2021). Given the considerable distance of 12 Mb between the sunburn and the impedance QTLs the loci do not overlap and annotations within the QTLs showing a direct connection to wax formation were not identified. Indirect interactions are a possible option, particularly regarding the reported differing ‘Shine Muscat’ waxy phenotype and identified wax-related genes on chromosome 11 (Zhang et al., 2021). In unaffected berries of Chardonnay (*V*IVC-No. 2455) the waxes had an intricately arranged platelet structure orientated perpendicular to the surface. With even the slightest symptoms of sunburn, these waxes have lost the crystalline structure and became relatively amorphous (Greer et al., 2006) indicating a dynamic system.

Completing, we have to note, that 20% of the annotated genes within the QTL regions are of unknown function and could play a role in any of the discussed or undiscussed potential sunburn mechanisms. For chromosome 10, four of them are even in the top 10 of highest expressed genes, which could be a reason for not noting all possible candidate genes.

### 4.4 Transferability to currently relevant cultivars

Differences in response to heat stress between varieties have long been known. Müller-Thurgau (1883) reported that the grapes of over 50 varieties were more or less affected by the heat event of 1883. Heating experiments with grapes in a metal box showed, which temperature levels are required to induce sunburn symptoms in different varieties. In particular, the early ripening varieties ‘Pinot Noir Précoce’ (PNP; *V*IVC-No. 9280) and ‘Malingre Précoce’ (MP; *V*IVC-No. 7249) were not affected in the field by the heat event of 1883, from which Müller-Thurgau deduced a maturity dependence. In contrast, in the present study a substantial sunburn damage in PNP (class 6 **Table 1**) was observed. This indicates the difficulties of reliable phenotyping of cultivars under the rather uncontrolled, multi-factorial and (formerly) rare natural sunburn events in the field. The commonly early ripening phenotype of PNP, MP, and CM is based on the *Ver1* locus (Frenzke et al., 2024) but does not affect sunburn resilience as reported above.

The rather uncontrolled field conditions result in differential ratings for the same cultivars, even under highly similar conditions in nearby plots as e.g. reported for ‘Pinot Noir’ (*V*IVC-No. 9279) that was rated with 5 in the national collection and 7 in the international collection within 100 m planting distance and under similar growing conditions, whereas clonal differences cannot be excluded. Within the F1-population, the rating for both plots correlate with 0.65 across all years, according to Pearson. This highlights the interaction between strong genetic predisposition and multi-factorial environmental influences. More controlled experiments could be helpful, but are still impacted by the preconditions of the grape samples. While berries of ‘Riesling Weiss’ (*V*IVC-No. 10077), ‘Silvaner Gruen’ (*V*IVC-No. 11805), ‘Elbling Weiss’ (*V*IVC-No. 3865), and ‘Pinot Noir’ were already damaged at 42°C in Müller-Thurgau´s metal box, PNP and MP were unaffected even under longer duration at 55°C. Müller-Stoll, 1939 reports Gutedel (‘Chasselas Blanc’; *V*IVC-No. 2473) to be very sensitive, while ‘Riesling Weiss’ and Traminer (‘Savagnin Blanc’; *V*IVC-No. 17636) also show damage, but to a less extend. In contrast, ‘Silvaner Gruen’ is described as relatively insensitive and remained unharmed under the respective conditions (Zschokke, 1931; Müller-Stoll, 1939). This can be confirmed by the recent damage ratings of those cultivars within the present study, as shown in **Table 1**. This broader set of cultivars, even though additional replicates of the observations made in 2019 are missing, can serve as an estimation of their individual sunburn resilience.

### 4.5 Knowledge transfer to breeding

The genetically based sunburn resilience in the investigated F1 population is mainly characterized by the two loci on chromosomes 10 and 11. As reported in **Figure 6**, the alleles in both loci have additive effects and the complete allelic pattern of the loci has to be considered to identify the genotypes with the best sunburn resilience. This makes transferability out of the population complex and a better understanding of underlying mechanisms and follow up experiments are necessary to develop suitable and reliable selection tools. Marker assisted selection (MAS) based on few linked SSR markers, as successfully applied for the introgression of resistance loci (Di Gaspero et al., 2012; Welter et al., 2007; Schwander et al., 2012; Töpfer and Trapp, 2022; Zendler et al., 2017) or to follow single-locus traits like berry color (Doligez et al., 2002; Röckel et al., 2020) or veraison (Zyprian et al., 2018; Frenzke et al., 2024) is not feasible for sunburn resilience yet.

The allele with the highest impact towards sunburn resilience in this population originates from ‘Subereux’ (chromosome 11), a resistance donor of the early French breeding affords. A number of 54 offspring are reported for this genotype in the *V*IVC database, where next to ‘Villard Blanc’ with 142 own offspring, most are unreleased breeding lines. As many of recent PIWI cultivars are based on this genepool, a broad set of possible crossing partners should be screened and exploited for further knowledge based breeding. In this QTL, the allele of ‘Bacchus Weiss’ assists the sunburn resilience, but the fact that ‘Bacchus Weiss’ itself is highly sensitive to sunburn (**Table 1**), underlines the complexity of this multi-factorial trait. Within the second QTL on chromosome 10, the most effective allele originates from ‘Seyval Blanc’; also a resistant French variety. This allele is accessible in 40 reported offspring and further developed breeding lines. Based on the knowledge gained, breeders can consider sunburn resilience as an additional trait, by choosing suitable crossing partners and by integration of the identified markers into their MAS pipelines.

## 5 Conclusion

The resilience of grapevine berries to sunburn damage is mostly genetically based. This is demonstrated by the investigated F1 population showing a broad segregation for sunburn damage and a heritability of 0.59 *h^2^* for sunburn resilience based on 5 years of data acquisition. When sunburn damage inducing heat stress conditions occurred, the further environmental impact as well as the ripening stage were found to be of minor importance. The resulting phenotype can be explained by the additive effects in allele combination within the two identified QTLs on chromosomes 11 and 10. Next steps should include further attempts to identify the underlying genes as well as the validation and establishment of MAS markers based on the gained knowledge to assist breeding. To bridge the gap until new varieties selected based on this knowledge reach the market, it can be recommended to cultivate some recent PIWI varieties that were found to have this desired trait already by coincidence, like ‘Calardis Blanc’, or to limit the damage by appropriate canopy management - as already described by Müller-Thurgau back in 1883.

## Data Availability

The datasets presented in this study can be found in online repositories. The names of the repository/repositories and accession number(s) can be found in the article/**Supplementary Material**.

## Supplementary material

Supplementary Table 1 Annotated genes under the LOD_max_^-1^ QTL-Intervals for sunburn on chromosomes 10 and 11 and corresponding RNA-expression studies.

Supplementary Table 2 QTL-analysis results, phenotypic, Sequences and positions of developed SSR markers.

Supplementary Table 3 Map-summary, coverage of PN40024 v5 and haplotype map of the parents.

Supplementary Table 4 Weather data.

Supplementary Figure 1 QQ-plot of the BLUP-adjusted means.

Supplementary Figure 2 Genetic map; Chromosomes 1-10.

Supplementary Figure 3 Genetic map; Chromosomes 11-19.
