# Supplementary figures and images for: Heat waves reveal additive genetic effects leading to sunburn resilience of grapevine berries
Source: Front Plant Sci. 2025 Jun 30;16:1533345. doi: 10.3389/fpls.2025.1533345 (PMC12256528; doi:10.3389/fpls.2025.1533345)

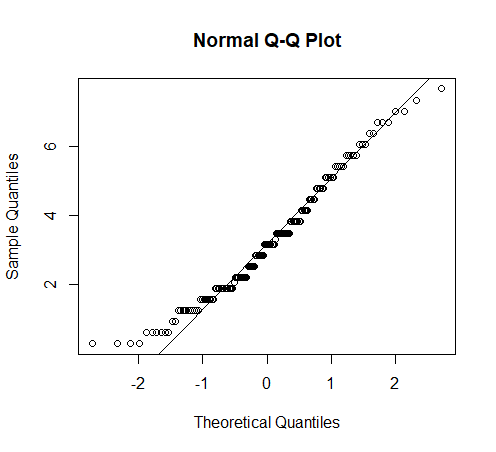

Supplement: Supplementary Figure 1 — QQ-plot of the BLUP-adjusted means. [file Image1.tiff]
